# Genetic Polymorphisms and Pesticide Exposure-Related Health Manifestations Among Grape Farmers in Maharashtra, India

**DOI:** 10.7759/cureus.108637

**Published:** 2026-05-11

**Authors:** Aishwarya Garud, Satyajeet Pawar, Kailas Datkhile, Pratik P Durgawale, Satish V Kakade, Satish R Patil

**Affiliations:** 1 Department of Molecular Biology and Genetics, Krishna Institute of Medical Sciences, Krishna Vishwa Vidyapeeth (Deemed to Be University), Karad, IND; 2 Department of Microbiology, Krishna Institute of Medical Sciences, Krishna Vishwa Vidyapeeth (Deemed to Be University), Karad, IND; 3 Department of Genetics, Krishna Institute of Science and Technology, Krishna Vishwa Vidyapeeth (Deemed to Be University), Karad, IND; 4 Department of Community Medicine, Krishna Institute of Medical Sciences, Krishna Vishwa Vidyapeeth (Deemed to Be University), Karad, IND

**Keywords:** dna repair genes, genetic polymorphism, occupation health, paraoxonase 1, pesticide exposure, pesticide toxicity

## Abstract

Background

Occupational exposure to pesticides has been associated with a range of adverse health outcomes among agricultural workers. Genetic polymorphisms in detoxification and DNA repair pathway genes may influence individual susceptibility to these effects. Grape farmers in southwestern Maharashtra, India, represent a high-risk occupational group facing routine and intensive pesticide exposure throughout the cultivation season, yet molecular epidemiological data from this population remain limited.

Methods

A cross-sectional exploratory study was conducted among 204 pesticide-exposed grape farmers from Sangli and nearby districts of southwestern Maharashtra, India. Demographic characteristics, exposure patterns, and self-reported symptoms experienced following pesticide exposure were recorded using a structured questionnaire. Oxidative stress markers, including malondialdehyde (MDA) and ferric reducing antioxidant power (FRAP), were measured as secondary biological indicators. Genotyping of paraoxonase 1 (*PON1*) polymorphisms (Q192R, L55M), apurinic/apyrimidinic endonuclease 1 (*APE1*), and X-ray repair cross-complementing proteins (*XRCC1*, *XRCC2*, and *XRCC3*) was performed using polymerase chain reaction-restriction fragment length polymorphism (PCR-RFLP). Associations between genotypes, exposure-related parameters, and self-reported symptoms were evaluated using chi-square tests and odds ratios (ORs) with 95% confidence intervals (CIs). A p-value ≤ 0.05 was considered statistically significant.

Results

The majority of participants were male, 177 (86.8%), with most falling in the 31-50 year age group, 110 (53.9%). A total of 170 (83.3%) reported daily exposure of ≥6 hours, and none reported use of personal protective equipment. The mean MDA was 5.24 ± 2.02 µmol/L, and the mean FRAP was 443.39 ± 250.90 µmol/L. Overall, 80 (39%) participants reported at least one symptom following pesticide exposure. Prolonged daily exposure (≥6 hours) was significantly associated with increased odds of symptom experience (OR = 3.5; 95% CI: 1.38-8.9; p = 0.006). *PON1* L55M heterozygosity showed a borderline significant association with increased likelihood of self-reported reactions (OR = 1.79; 95% CI: 0.99-3.2; p = 0.049). The *XRCC1* rs25487 variant genotype was inversely associated with symptom experience (OR = 0.25; 95% CI: 0.07-0.92; p = 0.034), though this finding requires cautious interpretation given the low variant cell count.

Conclusion

Prolonged daily pesticide exposure was independently associated with increased likelihood of self-reported health reactions. The borderline association of *PON1* L55M and the exploratory finding for *XRCC1* rs25487 represent preliminary signals requiring confirmation in larger studies with objective exposure assessment and validated clinical outcomes.

## Introduction

Pesticide use in agriculture has grown substantially in recent decades, particularly in developing countries such as India. The unregulated or excessive use of pesticides poses considerable health risks to agricultural workers. Farm workers are routinely exposed to pesticides through inhalation, dermal contact, and ingestion, leading to an array of acute and chronic health effects. Pesticide exposure is known to induce oxidative stress by generating reactive oxygen species and disrupting cellular homeostasis. Such exposure is associated with outcomes, including neurological, respiratory, dermatological, and systemic symptoms and reactions reported by exposed individuals [1].

The detoxification of pesticides in humans largely depends on the activity of paraoxonase 1 (*PON1*), an enzyme that hydrolyses toxic metabolites. Genetic polymorphisms in the *PON1* gene significantly influence its activity. In particular, the Q192R polymorphism affects the catalytic efficiency of the enzyme, whereas the L55M polymorphism influences its serum concentration [2]. These variations may alter an individual’s capacity to detoxify pesticides, thereby contributing to differences in susceptibility to pesticide-related health outcomes.

In addition, DNA repair mechanisms have a crucial role in the maintenance of genomic stability under conditions of oxidative stress. Genes including X-ray repair cross-complementing 1 (*XRCC1*), *XRCC2*, *XRCC3*, and apurinic/apyrimidinic endonuclease 1 (*APE1*) are involved in key repair processes, including base excision repair (BER) and homologous recombination. Polymorphisms in these genes may compromise DNA repair efficiency, leading to increased accumulation of damage and variability in individual response to pesticide exposure [3].

Taken together, genetic variability in both detoxification and DNA repair pathways may result in differential susceptibility to pesticide-induced health effects among exposed individuals.

Despite the high pesticide exposure burden in Indian agriculture, molecular epidemiological evidence exploring these associations remains limited. Particularly among grape farm workers of southwestern Maharashtra, who face routine and intensive pesticide exposure throughout the cultivation season. Grape cultivation is an intensive agricultural practice in India, involving repeated application of multiple chemical classes.

The primary objective of this study was to determine the prevalence of genetic polymorphisms in detoxification (*PON1 *L55M*, *Q192R) and DNA repair genes (*APE1, XRCC1, XRCC2, XRCC3*) among occupationally exposed grape farmers of southwestern Maharashtra, India. The secondary objective was to explore associations between these genetic variants and self-reported symptoms experienced following pesticide exposure. Oxidative stress markers were evaluated as supportive biological indicators of exposure burden.

The findings of this exploratory cross-sectional investigation are not intended to establish universal causal relationships or to be generalised beyond this population and region. Rather, this study contributes local epidemiological evidence from an understudied agricultural community, and the observed associations are presented as preliminary signals warranting investigation in larger, multi-site studies with more rigorous exposure quantification and validated clinical outcome measures.

## Materials and methods

Study design and population

The study protocol was approved by the Institutional Ethics Committee (approval number: 462/2022-2023). Written informed consents were obtained from all participants before enrolment.

This cross-sectional exploratory study was conducted among pesticide-exposed grape farmers of Maharashtra, India. The sample collection was carried out from January 2024 to December 2025 in the major grape-growing regions of Sangli and nearby districts. Individuals aged ≥18 years who were actively involved in pesticide handling, mixing, or spraying for at least a year were included. Participants with a history of chronic systemic illness were excluded.

Sample size calculation

The sample size was calculated considering the objective of pesticide exposure-associated health manifestations among farmers. According to Kori et al., adverse health effects had occurred to an average of 32.2% farmers [4]. Based on these findings, with a 95% confidence level and 7% absolute error (precision), the minimum required sample size was calculated to be 178 farmers using the formula: $n = \frac{Z^{2}pq}{l^{2}} = \frac{4 \times 32.2 \times 67.8}{7^{2}} = \frac{8721.84}{49} \approx 178$. However, in the present study, data from 204 pesticide-exposed grape farmers are presented.

A total of 220 individuals with an occupational pesticide exposure history were initially recruited. After data cleaning, 16 participants with incomplete or missing data were excluded. The final analysis included 204 participants, which exceeds the calculated minimum sample size and ensures adequate precision for prevalence estimation and exploratory association analyses.

Demographic data were collected using a structured questionnaire administered through face-to-face interviews. The questionnaire captured demographic characteristics (age, gender, education), lifestyle factors (dietary habits, tobacco consumption), occupational exposure history (duration of pesticide use and daily exposure time), family history of diseases, and self-reported symptom experience following pesticide exposure.

Pesticide exposure profile

Pesticide exposure was assessed using a structured questionnaire capturing duration of occupational exposure and daily exposure hours. Participants were categorised based on self-reported exposure characteristics, including daily exposure duration (one to five hours and ≥6 hours per day) and years of pesticide exposure (1-10 years and >10 years). These variables were used to characterise exposure patterns within the study population. Exposure assessment was based on self-reported information and was not validated using environmental or biomonitoring data (see Appendices).

Participants were also asked about the types of pesticides used and the use of personal protective measures. Farmers reported the use of a wide range of agrochemicals, including insecticides, fungicides, and mixed commercial formulations. Commonly used pesticides included organophosphates (chlorpyrifos, malathion, phorate, dimethoate, dichlorvos, quinalphos, acephate, and phosalone), carbamates (carbofuran), pyrethroids (lambda-cyhalothrin and permethrin), and fungicides (mancozeb, zineb, captan, folpet, boscalid, metrafenone, flutriafol, procymidone, and vinclozolin). These findings reflect real-world mixed exposure conditions characteristic of grape cultivation in this region.

Assessment of pesticide exposure-related health reactions

Self-reported pesticide-related health effects were assessed based on symptoms experienced following pesticide exposure. Participants reporting at least one symptom were classified as Reaction Present, while those reporting no symptoms were classified as Reaction Absent. These binary categories served as the primary outcome variable for association analyses. The individual symptoms reported by participants are described descriptively in the results. These outcomes represent subjective indicators of exposure-related health experiences and do not constitute clinically validated diagnoses or direct biological endpoints.

Genotyping assay

Peripheral blood (5 mL) was collected in an ethylenediaminetetraacetic acid (EDTA) tube under aseptic conditions. Genomic DNA was extracted using the manufacturer’s protocol (OMEGA E.Z.N.A.® Blood DNA Mini Kit, Omega Bio-Tek, USA).

Genotyping of selected polymorphisms in PON1 (Q192R, L55M) and DNA repair genes (*APE1, XRCC1, XRCC2,* and *XRCC3*) was accomplished using polymerase chain reaction-restriction fragment length polymorphism (PCR-RFLP). PCR amplifications were carried out in a total reaction volume of 25 µL containing genomic DNA, primers, deoxynucleotide triphosphates (dNTPs), magnesium chloride (MgCl_2_), buffer, and Taq DNA polymerase. Amplification conditions and fragment sizes were adopted from previously published studies [5-8]. Amplification of each gene fragment was carried out under optimised conditions with an initial denaturation at 95°C (five minutes), followed by 30-35 cycles of denaturation at 95°C (30 seconds), annealing at 55-58°C (20 to 30 seconds), and extension at 72°C (20-45 seconds), with a final extension at 72°C for 5 to 10 minutes.

Following amplification, the PCR products were subjected to restriction enzyme digestion specific to each polymorphism. The restriction enzymes used were BfaI for *APE1*, NciI for *XRCC1*, HphI for *XRCC2*, NlaIII for *XRCC3*, MboI for *PON1* (Q192R), and NlaII for *PON1* (L55M). The digested products were separated by agarose gel electrophoresis and visualised under ultraviolet illumination to determine genotype based on fragment patterns. To ensure genotyping accuracy, approximately 10% of samples were randomly selected and re-genotyped, yielding 100% concordance. Negative controls were included in each PCR run to monitor contamination. The primer sequences, PCR product length and digested band sizes for each polymorphism are summarised in Table 1.

**Table 1:** Primer sequences, PCR product length and RFLP FP: forward primer, RP: reverse primer, BP: base pair, W: wild, H: heterozygous, V: variant, PCR: polymerase chain reaction; RFLP: restriction fragment length polymorphism

Gene	Primer sequence	PCR product	RFLP bands(bp)
*APE1* (rs1130409)	FP:5’-CTG TTT CAT TTC TAT AGG CTA-3’	164 bp	W-164 H-164, 144 V-144
	RP:5’-AGG AAC TTG CGA AAG GCT TC-3’		
*XRCC1* (rs25487)	FP:5’-CAG TGG TGC TAA CCT AAT C3’	871 bp	W-461, 278,132 H-593, 461, 278, 132 V-593, 278
	RP:5’- AGT AGT CTG CTG GCT CTG G3’		
*XRCC2* Arg188His rs3218536	FP:5’-AGT TGC TGC CAT GCC TTA CA3’	290 bp	W-290 H-290,148, 142 V-148, 142
	RP:5’- TGTAGTCACCCATCTCTCTGC3’		
*XRCC3 *(Thr241Met, rs861539)	FP:5’-GGT CGA GTG ACA GTC CAA AC3’	455 bp	W-315 H-315, 210, 140, 105 V-210, 140, 105
	RP:5’- TGCAACGGCTGAGGGTCTT3’		
*PON1* Q192R, rs662	FP:5’-GGG ACC TGA GCA CTT TAT GGC 3'	176 bp	W-145, 31 H-145, 117, 31,28 V-117, 31, 28
	RP:5’-CAT CGG GTG AAA TGT TGA TTC C 3'		
*PON1* rs854560, L55M	FP:5’-TTG AGG AAT AAG CTC TAG TCC A 3'	384 bp	W-384 H-384, 282, 102 V-282,102
	RP:5’-GAA AGA CTT AAA CTG CCA GTC C3’		

Biochemical analysis

Blood serum was separated by centrifugation and used for oxidative stress assessment. Malondialdehyde (MDA) levels were measured using the thiobarbituric acid reactive substances (TBARS) assay as an index of lipid peroxidation. The ferric reducing antioxidant power (FRAP) assay was performed to assess total antioxidant capacity (TAC). MDA and FRAP assays for all samples were performed following standard protocols [9,10].

All biochemical assays were performed under standardised laboratory conditions. Samples were processed in batches with consistent reagent lots and protocols to minimise inter-assay variability.

Statistical analysis

Categorical variables were expressed in frequencies and percentages, while continuous variables were expressed as mean ± standard deviation. Associations between genetic polymorphisms and pesticide-related reactions were assessed using the chi-square test and odds ratios (ORs) with 95% confidence intervals (CIs). A p-value < 0.05 was considered statistically significant. Hardy-Weinberg equilibrium (HWE) was assessed in the study population using the chi-square test to evaluate genotype distribution.

## Results

Demographic characteristics

A total of 204 pesticide-exposed farmers were included in the study (Table 2). The majority of participants were male (177, 86.8%) and aged 31-50 years (110, 53.9%). Most participants had an education below higher secondary level (135, 66.2%) and reported mixed dietary habits (199, 97.5%). Tobacco consumption was reported by 63 participants (30.9%), while 47 (23.0%) had a family history of diseases. A large proportion of farmers reported prolonged pesticide exposure, with 170 (83.3%) exposed for more than ≥6 hours per day and 122 (59.8%) having more than 10 years of exposure.

**Table 2:** Demographic frequencies in the study population HSC: higher secondary certificate

Variable	Category	Participants (n = 204)
Age group	18-30 years	61 (29.9%)
	31-50 years	110 (53.9%)
	>51 years	33 (16.2%)
Gender	Male	177 (86.8%)
	Female	27 (13.2%)
Education	Below HSC	135 (66.2%)
	Above HSC	69 (33.8%)
Diet	Vegetarian	5 (2.5%)
	Mixed	199 (97.5%)
Tobacco consumption	Yes	63 (30.9%)
	No	141 (69.1%)
Family history of disease	Yes	47 (23.0%)
	No	157 (77.0%)
Type of disorder	Metabolic	31 (15.2%)
	Non-metabolic	30 (14.7%)
	Both	4 (2.0%)
	None	139 (68.1%)
Daily exposure hours	1-5 hours	34 (16.7%)
	≥6 hours	170 (83.3%)
Years of pesticide exposure	1-10 years	82 (40.2%)
	More than 10 years	122 (59.8%)

Pesticide usage pattern

The study population reported extensive use of multiple classes of pesticides, with organophosphate insecticides being the most frequently used group. Fungicides such as mancozeb and zineb were also widely used due to their relevance in grape cultivation. Many farmers reported using multiple pesticides simultaneously or sequentially, often without standardised dosing or adequate protective measures. Also, none of the participants mentioned the use of personal protective gear while handling pesticides.

**Figure 1:**
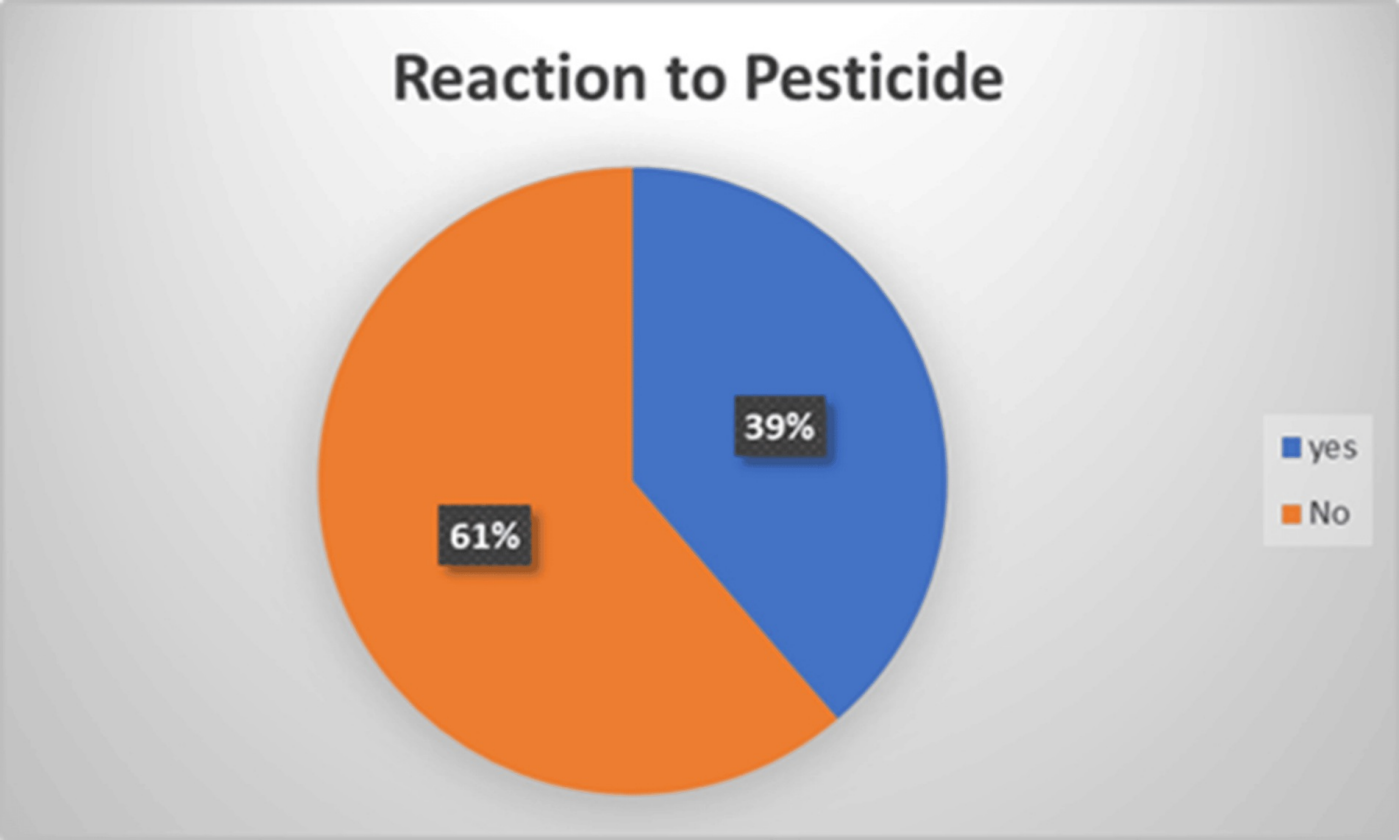
Frequency of self-reported reactions to pesticides following exposure among farmers

Among the 204 pesticide-exposed participants, 39% (n = 80) reported at least one symptom following pesticide exposure, as shown in Figure 1. The most frequently reported symptom category was cold, sneezing, and upper respiratory irritation (n = 28), followed by skin-related complaints, including itching, rashes, burning sensation, and allergy (n = 23). Breathing problems or breathlessness were reported by 11 participants, as was eye irritation, itching, and redness (n = 11). Systemic symptoms, including weakness, body pain, weight loss, and bone pain, were reported by nine participants. Neurological symptoms comprising dizziness, tremors, confusion, and memory or balance disturbances were similarly reported by nine participants. Gastrointestinal complaints, including stomachache, nausea, vomiting, and acidity, were noted in six participants, while fever or feverish feeling and swelling were each reported by five participants.

Oxidative stress profile

The mean MDA level among the study participants was 5.24 ± 2.02 µmol/L, while the mean of FRAP values was 443.39 ± 250.90 µmol/L.

Genotype distribution

**Figure 2:**
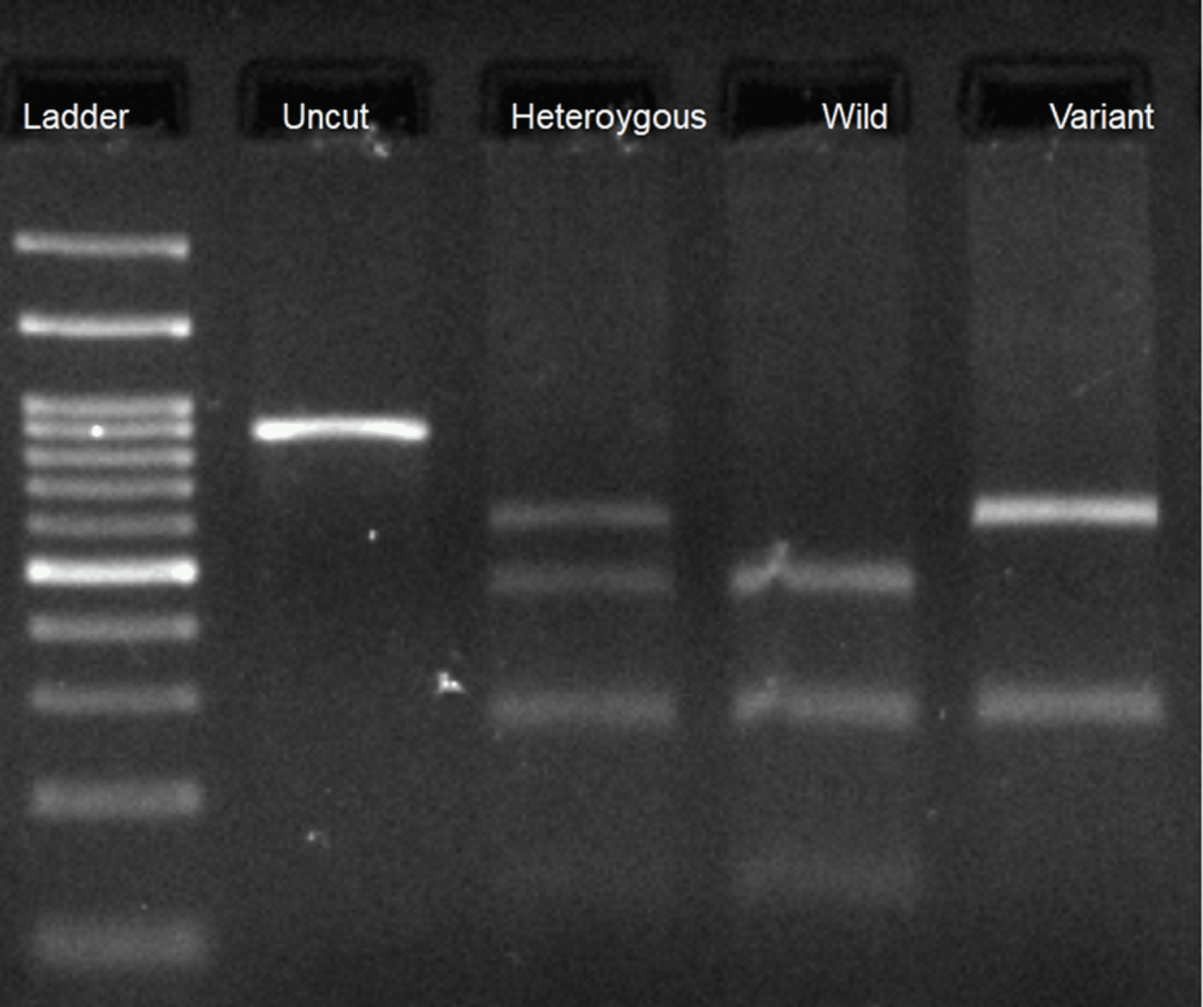
Agarose gel image showing XRCC1 polymorphism Lane 1: 100-bp ladder, Lane 2: undigested polymerase chain reaction (PCR) product (band at 871 bp), Lane 3: heterozygous genotype (bands at 593 bp, 461 bp, 278 bp, and 132 bp), Lane 4: wild genotype (bands at 461 bp, 278 bp, and 132 bp), Lane 5: variant genotype (bands at 593 bp and 278 bp)

**Figure 3:**
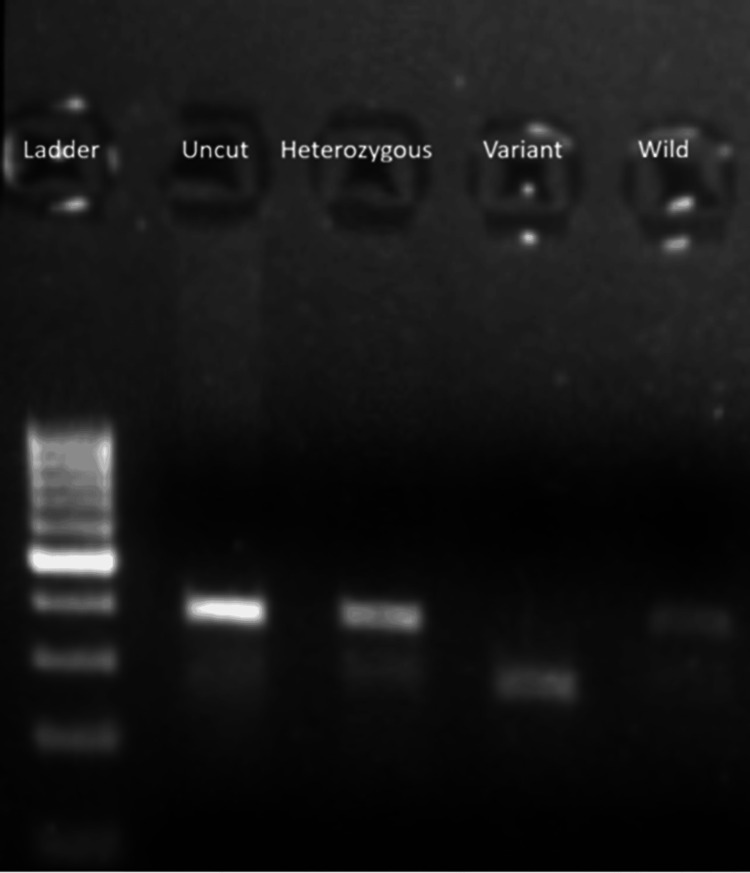
Agarose gel image showing PON1 polymorphism Lane 1: 100 bp DNA ladder, Lane 2: undigested polymerase chain reaction (PCR) product (band at 384 bp), Lanes 3: Heterozygous genotype (bands at 384, 282, and 102 bp), Lane 4: variant genotype (bands at 282 and 102 bp), Lanes 5: wild genotype (bands at 384bp)

Representative PCR-RFLP gel images confirming genotype patterns for *XRCC1* and *PON1* (L55M) are shown in Figures 2-3, respectively. Overall, the wild genotype was the most frequent across all studied polymorphisms. A considerable proportion of the study population exhibited heterozygous genotypes, particularly in DNA repair genes. *XRCC1*, *XRCC2*, and *XRCC3* confirmed to have HWE (p >0.05), whereas *APE1* and *PON1* (L55M and Q192R) polymorphisms deviated from HWE (p < 0.05). *APE1* heterozygosity was observed in 88 participants (43.1%), *XRCC1* heterozygosity in 79 participants (38.7%), *XRCC2* heterozygosity in 35 participants (17.2%), *XRCC3* heterozygosity in 65 participants (31.9%) and was also notable for *PON1* L55M (80 (39.2%)) and *PON1* Q192R (72 (35.3%)). While variant (homozygous mutant) genotypes were less frequent across most loci. Among DNA repair genes, the heterozygous variant genotype *APE1* was observed in 88 individuals (43.1%), the *XRCC2* variant genotype was observed in two participants (1.0%), the *XRCC3* heterozygous genotype was observed in 65 individuals (31.9%) and the *XRCC1* in 17 (8.3%). For *PON1* polymorphisms, the variant genotype was less frequent in L55M (4 (2%)) compared with Q192R (29 (14.2%)) (Table 3).

**Table 3:** Genotypic frequency distribution in the study population

Gene	Genotype	Participants (n = 204)
APE1_164BP	Wild	113 (55.4%)
	Heterozygous	88 (43.1%)
	Variant	3 (1.5%)
XRCC2-290BP	Wild	167 (81.9%)
	Heterozygous	35 (17.2%)
	Variant	2 (1%)
XRCC1-871BP	Wild	108 (52.9%)
	Heterozygous	79 (38.7%)
	Variant	17 (8.3%)
XRCC3-455BP	Wild	130 (63.7%)
	Heterozygous	65 (31.9%)
	Variant	9 (4.4%)
PON1_L55M	Wild	120 (58.8%)
	Heterozygous	80 (39.2%)
	Variant	4 (2%)
PON1_176BP	Wild	103 (50.5%)
	Heterozygous	72 (35.3%)
	Variant	29 (14.2%)

Daily duration of pesticide exposure was significantly associated with self-reported reactions following pesticide exposure (Table 4). Individuals exposed to pesticides for six or more hours per day had significantly higher odds of reporting reactions experienced post-pesticide application compared to those with one to five hours of exposure per day (OR = 3.5; CI: 1.38-8.9; p = 0.006).

**Table 4:** Association between daily duration of pesticide exposure and and self-reported symptom experience following pesticide exposure * Fisher's exact test applied due to low expected cell frequency.

	Duration	Reaction present	Reaction absent	Chi-square	OR (95%CI)	P-value
Daily exposure hours	1-5 hours	6 (7.6%)	28 (22.4%)	7.64	1	-
	6 or more	73 (92.4%)	97 (77.6%)		3.5 (1.38-8.9)	0.0064*

Analyses of all six polymorphisms in association with self-reported symptoms experienced post-pesticide exposure were performed. However, genetic polymorphism in *PON1* (L55M) and *XRCC1* (rs25487) showed association patterns with post-exposure related reactions (Table 5). Individuals with a *PON1* heterozygous genotype had higher odds of having a reaction to pesticide (OR = 1.79; CI = 0.99-3.2; p = 0.049) than the wild genotype, but this was borderline significant with a broad CI. A variant of PON1 also suggested a similar trend but did not reach statistical significance. *XRCC1* variant genotype having low cell count was negatively associated with self-reported reactions experienced after pesticide exposure, when compared to wild genotype (OR = 0.25; CI = 0.07-0.92; p = 0.034). While the heterozygous genotype indicated the same pattern but did not reach statistical significance.

**Table 5:** Association between genetic polymorphisms and self-reported reaction post-pesticide exposure * Fisher's exact test applied due to low expected cell frequency.

Gene	Genotype	Reaction present	Reaction absent	Chi-square	p-value	OR (95%CI)	p-value
PON1_L55M	Wild	39 (49.4%)	81 (64.8%)	6.09	0.048	(1)	-
	Heterozygous	37 (46.8%)	43 (34.4%)			1.79 (0.99-3.2)	0.049
	Variant	3 (3.8%)	1 (0.8%)			6.23 (0.62-61.9)	0.112*
XRCC1 (rs25487)	Wild	50 (63.3%)	58 (46.4%)	6.92	0.031	(1)	-
	Heterozygous	26 (32.9%)	53 (42.4%)			0.569 (0.3-1.04)	0.066
	Variant	3 (3.8%)	14 (11.2%)			0.25 (0.07-0.92)	0.034*

## Discussion

The present study was conducted as an exploratory, cross-sectional investigation among grape farmers of southwestern Maharashtra. An occupationally exposed population that has received limited molecular epidemiological attention despite facing routine and intensive pesticide exposure throughout the cultivation season. The primary aim was to characterise the prevalence of genetic polymorphisms in detoxification (*PON1* L55M, Q192R) and DNA repair genes (*APE1*, *XRCC1, XRCC2, XRCC3*), and to explore their associations with self-reported symptoms experienced following pesticide exposure. The findings represent hypothesis-generating observations within this specific cohort and are not proposed as universally generalised conclusions.

The spectrum of self-reported symptoms following pesticide exposure observed in this study spanned multiple physiological domains and was preliminary and subjective in nature, without clinical validation. Nevertheless, the pattern of symptoms reported is consistent with the well-documented multi-system toxicity of organophosphates, carbamates, fungicides, and other agrochemical classes predominantly used in this setting [11]. The overall prevalence of self-reported pesticide-related reactions (39%) is comparable to previously reported agricultural populations [12]. Notably, none of the participants reported use of personal protective equipment during pesticide spraying, reflecting real-world exposure conditions and further highlighting the occupational vulnerability of this workforce. Similar findings of no safety knowledge are mentioned by Kori et al. [4].

The mean MDA level observed in this study (5.24 ± 2.02 µmol/L) may reflect increased lipid peroxidation, consistent with previous evidence demonstrating a positive correlation between pesticide metabolite concentrations and oxidative stress biomarkers, including MDA in occupationally exposed male farmers [13]. Similarly, FRAP ranged from 443.39 ± 250.90 µmol/L throughout the population. A systematic review on TAC as a biomarker of pesticide exposure reported that declining TAC levels are consistently associated with pesticide exposure, irrespective of chemical nature, with FRAP emerging as the most frequently applied and relatively consistent assay across studies [14]. However, both MDA and FRAP as biomarkers have certain limitations, as variability in analytical methods and sample handling has been reported to influence its measurement and interpretation across laboratories [15]. A notable limitation of the present study is the absence of an internal unexposed control group from the same population and laboratory setting, which would have allowed a more valid contextual comparison. Future studies should incorporate matched unexposed controls to better characterise the oxidative burden attributable specifically to pesticide exposure. These oxidative stress findings are therefore presented as supportive biological indicators of exposure burden rather than definitive mechanistic evidence.

Recent large-scale evidence highlights that real-world pesticide exposure occurs as complex chemical mixtures rather than isolated compounds [16]. In the present study, prolonged daily pesticide exposure (≥6 hours/day) was significantly associated with a higher likelihood of reporting pesticide-related health reactions. A systematic review and meta-analysis on insecticide exposure reported elevated odds of respiratory symptoms and asthma among occupationally exposed populations, supporting a dose-response relationship between exposure duration and respiratory health outcomes [17].

Beyond respiratory effects, occupational pesticide exposure is recognised to elicit a broad spectrum of acute health manifestations, including dizziness, nausea, headache, skin irritation, and respiratory discomfort. The heterogeneity in reported symptoms across studies is attributable to several factors, including variations in exposure duration, cumulative dose, chemical class of pesticide used, individual susceptibility, and the availability and consistency of personal protective equipment use [18]. A limitation of the present study is its reliance on self-reported symptom data rather than clinically validated diagnostic outcomes. Nevertheless, the observed association between prolonged exposure duration and increased symptom reporting is consistent with the broader occupational health literature, which documents a positive relationship between pesticide exposure and the reporting of health symptoms in agricultural workers. Future research incorporating objective clinical assessments, biomarker quantification, and longitudinal follow-up would be necessary to establish causal relationships and to better characterise the dose-response dynamics of pesticide-related health effects in this population.

Among the six polymorphisms examined, two showed associations with self-reported symptom experience. Individuals carrying the *PON1* L55M heterozygous genotype had higher odds of reporting pesticide-related reactions compared to those with the wild genotype (OR = 1.79; 95% CI: 0.99-3.2; p = 0.049), though this association was borderline significant with wide CIs. The biological plausibility of this finding is supported by existing evidence. A significant decrease in mean *PON1* enzyme activity from LL to LM to MM genotypes has been observed in human serum, suggesting that even heterozygous carriers may experience a partial reduction in detoxification capacity [19]. In the present study, the population of grape farm workers from southwestern Maharashtra, the *PON1* L55M genotype distribution revealed that 39.2% of participants carried the heterozygous (LM) genotype and 2% carried the variant homozygous (MM) genotype. This relatively high proportion of heterozygous individuals within the study population is noteworthy, as the LM genotype is associated with intermediate *PON1* enzyme activity compared to the wild-type LL genotype.

The *XRCC1* variant genotype (rs25487, Arg399Gln) was negatively associated with self-reported pesticide-related health reactions (OR = 0.25; 95% CI: 0.07-0.92; p = 0.034). However, this finding must be interpreted with considerable caution, given the low variant genotype count in the present study, and replication in a larger sample is necessary before drawing any conclusions. *XRCC1* is a scaffolding protein that plays a central role in the BER pathway, facilitating the repair of DNA strand breaks and base damage induced by oxidative stress and exogenous genotoxic agents. The rs25487 G>A polymorphism change has been associated with reduced DNA repair capacity in pesticide-exposed agricultural workers [20]. Individuals carrying the variant allele would therefore theoretically be expected to exhibit greater genotoxic damage accumulation rather than protection from acute health reactions. Acute self-reported symptoms following pesticide exposure are predominantly mediated through cholinesterase inhibition, systemic toxicity, and metabolic pathways, rather than through DNA repair efficiency, which operates over a longer biological time frame. Furthermore, no significant associations were identified for *APE1*, *XRCC2*, or *XRCC3* polymorphisms, consistent with the expectation that DNA repair genes exert effects over a longer timescale. Taken together, these findings suggest that DNA repair gene polymorphisms are unlikely to be informative markers of acute pesticide-related symptom burden, and their relevance in this population warrants evaluation in studies designed specifically to assess long-term genotoxic outcomes.

The present study has certain limitations inherent to its field-based, cross-sectional design. Exposure assessment and health outcome data relied on self-reported information, introducing the possibility of recall bias, and the modest sample size limited the scope for multivariate adjustment and subgroup analyses. The absence of an unexposed control group and lack of biomarker-based exposure validation are acknowledged, and future studies should address these through longitudinal designs incorporating objective biomonitoring and matched controls. Notwithstanding, this study provides valuable preliminary molecular epidemiological evidence from an understudied agricultural community, and the associations identified warrant further investigation in larger confirmatory studies.

## Conclusions

This exploratory study provides preliminary molecular epidemiological evidence from an understudied grape-cultivating population of southwestern Maharashtra. Prolonged daily pesticide exposure (≥6 hours/day) was independently associated with increased likelihood of self-reported pesticide-related health reactions. Among the six polymorphisms examined, *PON1* L55M heterozygosity showed a borderline association with increased symptom experience, while the *XRCC1* rs25487 variant genotype showed an unexpected inverse association that could not be meaningfully interpreted given the low variant count. DNA repair gene polymorphisms as a whole did not emerge as informative markers of acute symptom burden in this cohort. The absence of personal protective equipment use across the entire study population further underscores the occupational vulnerability of this workforce. These hypothesis-generating findings warrant confirmation in larger longitudinal studies incorporating matched unexposed controls, objective biomonitoring, and validated clinical outcomes to better characterise the role of genetic and environmental factors in pesticide-related health risk among agricultural communities.

## Appendices

Study of single nucleotide polymorphisms in genes associated with DNA repair, carcinogen detoxification and metabolic pathway in farmers with pesticide exposure

(This questionnaire was developed and used under the above IEC-approved PhD study)

A) Administrative Information

(For identification and follow-up purposes only and are not reported in the study)

1) Name of Participant: __________________________________________________

2) Address: __________________________________________________

3) Mobile Number: ______________________________

B) Demographic Information

4) Date of Birth: _______________________ Age: __________

Gender: Male/Female/Other

5) Education: Below HSC/Above HSC/Other: ______________________________

6) Dietary Habits: Vegetarian/Non-vegetarian/Mixed

7) Tobacco Consumption: Yes/No

8) Family History of Diseases: Yes/No

If Yes - Type of Disorder___________________________

□ Metabolic □ Non-Metabolic □ Both

C) Occupational Pesticide Exposure History

9) Duration of Pesticide Use (Years of Occupational Exposure):

□ 1-10 years

□ More than 10 years

10) Daily Exposure Duration (hours spent handling/spraying pesticides per day):

□ 1-5 hours

□ 6 or more hours

11) Types of Pesticides/Agrochemicals Used (please specify): __________________________________________________________________________________________

12) Use of Personal Protective Equipment (PPE) during pesticide handling/spraying: Yes/No

If Yes - specify type(s): _______________________________________________

D) Self-Reported Health Reactions Following Pesticide Exposure

13) Have you experienced any health symptoms or reactions after handling or spraying pesticides? Yes/No

If yes - please describe the symptoms in your own words:

_________________________________________________________________________________________________________________________________________________________________________________________________________________________________

Interviewer's Signature: ___________________________

Date: _____________________

Participant's Signature/Thumb Impression: ___________________________
